# College and Hospital Notes

**Published:** 1908-01

**Authors:** 


### COLLEGE AND HOSPITAL NOTES.

The Board of Directors of the Hospital for Deformities and Joint Diseases sent an invitation to the Journal to attend the dedication of the opening of the recent addition to the present Hospital, 1917 Madison Avenue, between one hundred and twenty-third and one hundred and twenty-fourth streets, New York. The exercises were held Saturday afternoon, November 9, between the hours of two and six o'clock. It is a matter of regret that we are unable to attend.

The Hahnemann Hospital, which is the new name for the Buffalo Homeopathic Hospital, has purchased a fine site, at the corner of Linwood and Lafayette Avenues. The trustees of the reorganised institution paid about $25,000 for the property which was owned by Andrew Langdon and F. L. C. Atherton. The site is composed of three lots with depths of about 300 feet and an aggregate frontage of 300 feet on Linwood avenue. Building will probably begin in the spring. The trustees are raising a fund of $100,000 for the new institution.

The Marine Hospital, which has been noticed in these columns several times as very nearly an accomplished fact, at last seems to be getting a fair start. A press despatch from Washington a few days ago said that, the Treasury department will advertise for bids for the construction of the Marine Hospital in Buffalo soon after the first of January. The plans have been redrawn to secure bids within the $90 000 available.

